# INAHTA member agency stories of engaging, adaptable, and impactful HTA

**DOI:** 10.1017/S0266462324004732

**Published:** 2024-12-10

**Authors:** Sophie Söderholm Werkö, Sylvie Bouchard, Erni Z. Romli, Chunmei Li, Li-Ying Huang, Charlotte Pelekanou, Lauren Elston, Tara Schuller

**Affiliations:** 1 The Swedish Agency for Health Technology Assessment and Assessment of Social Services (SBU), Stockholm, Sweden; 2 Institut national d’excellence en santé et en services sociaux, Québec, QC, Canada; 3 Malaysian Health Technology Assessment Section, Ministry of Health Malaysia, Putrajaya, Malaysia; 4 Ontario Health, Toronto, ON, Canada; 5 Centre for Drug Evaluation, Taiwan, Republic of China; 6 National Institute for Health and Care Excellence, Manchester, United Kingdom; 7 Health Technology Wales, Cardiff, United Kingdom; 8 Institute of Health Economics, Edmonton, AB, Canada

**Keywords:** technology assessment, biomedical, decision making, health care quality, access, evaluation

## Abstract

Health technology assessment (HTA) agencies assess evidence to support decision making about which technologies to provide and pay for in the health system. HTA impact is understood as the influence that HTA report findings can have in the health system, including impacts on reimbursement decisions, changes to health outcomes, or broader system or societal impacts. The International Network of Agencies for Health Technology Assessment (INAHTA) is a global network of publicly funded HTA agencies. INAHTA’s mission, in part, is to advance the impact of HTA to support reimbursement decisions and the optimal use of health system resources. Each year, INAHTA awards the David Hailey Award for Best Impact Story to the member agency that shares the best story, as voted by fellow members, about HTA impact. The impact story sharing program in INAHTA contributes to a deeper understanding of what works well (or not so well) in achieving HTA impact. This paper provides six impact stories from agencies that were finalists for the 2021 and 2022 David Hailey Impact Award for Best Impact Story: the Institut national d’excellence en santé et en services sociaux, the Malaysian Health Technology Assessment Section, Ontario Health, the Center for Drug Evaluation, the National Institute for Health and Care Excellence, and Health Technology Wales. These stories demonstrate that HTA agencies can, in differing ways, effectively support governments in their efforts to place evidence at the centre of decision making.

## Introduction

Health technology assessment (HTA) agencies have the challenging role of assessing evidence to support decision making about which technologies to provide and pay for in the health system. HTA reports are typically used in multistakeholder environments to provide evidentiary support for decisions about health technology reimbursement and use.

HTA impact is understood as the influence or impact of HTA report findings on decision making or outcomes at various points in the health system. Impacts may be observed not only in the use of the HTA report by particular decision makers but also in the changes observed in clinical practice, patient outcomes, or more broadly in society such as the utilization of social services, which result from decisions that are made based on the HTA. HTA can have a broad range of impacts that improve health system quality and sustainability, although establishing causal links with further downstream effects can be challenging. (1;2)

The International Network of Agencies for Health Technology Assessment (INAHTA) is a network of fifty-four publicly funded HTA agencies from thirty-three countries (2024). Part of INAHTA’s mission is to bring leadership and expertise to advance the science, practice, and impact of HTA. By better understanding how HTA impact is achieved, INAHTA contributes to better uptake and use of this important policy tool to support health system optimization and sustainability. (3) INAHTA has long recognized the importance of measuring and demonstrating the impact of HTA as one of the top ten important challenges facing HTA agencies. (4)

At the annual INAHTA Congress, INAHTA members share stories of HTA impact from their agencies to compete for a chance to win the *David Hailey Award for Best Impact Story.* This is an award granted by fellow members of INAHTA, who listen to the impact stories and select the best one to receive the *David Hailey Impact Award.* This collegial competition has been running since 2015, with the intent of fostering a deeper shared understanding about what works well (or not so well) in achieving HTA impact. (5)

This paper provides a summary of six impact stories that were selected as finalists for the 2021 and 2022 *David Hailey Impact Award.* It continues INAHTA’s earlier publications of impact stories (6;7) and describes how different HTA agencies around the globe have been successful in achieving HTA impact. The stories hail from Asia, North America, and Europe and refer to different technologies such as genome-wide sequencing, advanced cancer therapy, and surgical techniques. Some stories describe successes in preventing complications from coronavirus disease 2019 (COVID-19) symptoms as well as countering the negative effects of misinformation during the pandemic.

The stories that follow are authored by representatives of the six INAHTA member agencies that were finalists in the competition, namely: the Institut national d’excellence en santé et en services sociaux (INESSS) in Québec, Canada; the Health Technology Assessment Section, Ministry of Health Malaysia (MaHTAS); Ontario Health (OH) in Ontario, Canada; the Center for Drug Evaluation (CDE), Taiwan, Republic of China; the National Institute for Health and Care Excellence (NICE); and, Health Technology Wales (HTW) in the United Kingdom.

## Story 1: Facilitating Paxlovid^MD^ integration, clinical use and real-world evidence monitoring in Quebec

At the end of 2021, midway between the fourth and fifth waves of COVID-19, officials in the healthcare system in Quebec were surprised by the transmission speed and magnitude of the Omicron variant. Although vaccination was well underway in the province, Quebec was facing difficulties in the supply of neutralizing antibodies and remdesivir, and intrahospital administration was almost impossible due to the risk of contamination and the lack of staff.

It was also difficult for SARS-CoV-2 positive outpatients at high risk of developing COVID-19 complications to make informed clinical decisions because only limited quantities of the few existing treatment options were available. Furthermore, uncertainties remained regarding the effectiveness of existing treatments against emerging variants. There was a dire need for access to medicines that could reduce hospitalizations and deaths. Fortunately, preliminary efficacy results were disclosed that convinced the U.S. Food and Drug Administration (FDA) to authorize nirmatrelvir/ritonavir (Paxlovid^**MD**^) for use in high-risk outpatients. That decision put pressure on the Canadian drug regulator, Health Canada, to conduct a priority assessment.

INESSS worked very closely with Health Canada and Pfizer representatives to obtain an aligned review process (i.e., of HTA review by INESSS with the regulatory review by Health Canada) enabling access to the latest available clinical data.

With confidence, the INESSS COVID-19 special team, supported by a group of experts working closely together since the beginning of the pandemic, worked relentlessly over the winter holiday break preparing for the issuance of Health Canada’s notice of compliance expected in early 2022. As such, INESSS was ready for the arrival of Paxlovid^**MD**^.

On January 18, INESSS published a preliminary clinical position. (8) In that paper, INESSS defined, according to current knowledge, the categories of patients who should be prioritized to receive Paxlovid^**MD**^, taking into account ethical concerns about limited product availability that could be a barrier to access by patients who could benefit from treatment. At the same time, INESSS released the original version of a clinical tool (9) that included a description of the laboratory tests to be done before and after initiating Paxlovid^**MD**^ along with a description of the drug and the alternative treatment options.

To guide prescribers in determining patient eligibility to receive the drug, at the end of January, INESSS produced a prescription template (10) to facilitate and standardize prescription writing and data collection.

Lastly, in mid-March, INESSS posted a webinar recording on the agency’s website that addressed frequently asked questions, and clinicians (doctors and pharmacists) could obtain continuing education credits by sending their professional associations the answers to a few questions.

The Government of Quebec chose to make Paxlovid^**MD**^ accessible by creating a program enabling all outpatients at risk of developing COVID-19 complications to receive the antiviral free of charge. Patients could receive their treatment at a pharmacy, regardless of whether they had private or public insurance. This implementation model facilitated the creation of a retrospective cohort of people treated with Paxlovid^**MD**^. Patient information was compiled from different clinical-administrative databases of the Ministry of Health and Social Services (MSSS) and the Régie de l’assurance maladie du Québec (RAMQ), and this was compared to a control group in the same retrospective cohort of persons with a positive test for COVID-19 receiving no treatment.

With this data, INESSS could describe the characteristics of patients who had been treated with Paxlovid^**MD**^ in Quebec; the proportion of individuals hospitalized from all causes and those whose hospitalization was due to COVID-19, and to assess the relative risk of hospitalization in individuals who have been treated with Paxlovid^**MD**^ compared to the control group.

From March 15 to June 12, 2022, 7,726 patients received Paxlovid^**MD**^ in Quebec. Pharmacists represented, by far, the most frequent prescribers. This was not a surprise; in addition to being the access point for medications, the pharmacists were well equipped to conduct a profile analysis for the patient and to manage drug interactions. The results of the comparative cohort study showed that Paxlovid^**MD**^ treatment was associated with a significantly reduced risk of hospitalization among incompletely primarily vaccinated high-risk outpatients and among completely vaccinated high-risk outpatients aged 70 years and older regardless of the time since the last dose of the vaccine. The study suggested that Paxlovid^**MD**^ may have reduced the risk of all-cause hospitalization in immunosuppressed people, although the statistical power of these analyses is insufficient to draw firm conclusions.

As of October 2023, INESSS updated its living literature review on different therapies and maintained discussions with many stakeholders to monitor the impacts of Paxlovid^**MD**^ in the Quebec population.

## Story 2: HTA riding out the storm an insight story – MaHTAS’ defining role in the face of the COVID-19 infodemic in Malaysia

When the COVID-19 pandemic spread to Malaysia, the local health system faced challenges on an unprecedented scale to manage the crisis. The COVID-19 pandemic experience made a significant impact in demonstrating the crucial national role of the Malaysian Health Technology Assessment Section (MaHTAS).

In 2020, MaHTAS experienced a sudden increase in requests for rapid assessment of health technologies, quadruple the amount of the pre-COVID-19 era. The requests, which came to MaHTAS from officials within the Ministry of Health such as the Director General of Health, Health Minister, and hospital directors, were received through various communication channels, mostly via digital platforms (69 percent, 279 requests). In rapid response, MaHTAS assessed various COVID-19 health technologies, including screening and diagnostic technologies (20.3 percent, fifteen reviews), clinical management techniques (36.5 percent, twenty-seven reviews), public health interventions (12.2 percent, nine reviews) as well as disinfection and sterilization technologies (31.0 percent, twenty-three reviews). Most of these assessments were generated within one week, including some within 24 hours, in order to inform decision makers regarding various COVID-19 management issues pertaining to procurement, clinical management, hospital preparedness, infection control and prevention, innovation investment, and “infodemic” (i.e., the epidemic of misinformation in particular) management.

Perhaps the most significant and impactful role MaHTAS played was in reducing the influence of the infodemic on health behaviors during the pandemic. The ubiquitous word-of-mouth communications and widespread reliance on social media facilitated an uncontrolled spread of misinformation. The highly damaging social perception and response to the misinformation disrupted the arduous efforts of the Malaysian government to manage the COVID-19 pandemic.

The pandemic presented MaHTAS with an opportunity to further demonstrate the usefulness of HTA to counter misinformation, as illustrated in the MaHTAS review of noncontact infrared thermometers (NCIT) (11) and disinfection tunnels (12). Among nonpharmacological interventions, NCITs were used as in screening for COVID-19. A rumor emerged claiming that the use of infrared laser from the thermometer could cause a brain tumor. This misinformation “went viral” across social media to the extent that many Malaysians accepted it as fact and refused NCIT screening. In response, a 24-hour rapid review was produced by MaHTAS that was referenced in a media press statement by the Malaysian Director-General of Health to rebut the rumor and gain public trust. (13).

As the COVID-19 pandemic unfolded, the business community seized opportunities to develop innovations intended to help tackle the pandemic; however, some product developers capitalized on the public fear of the virus with unproven interventions. For example, an aggressive campaign to promote disinfection tunnels for sterilization and disinfection in COVID-19 management misled the public. (14;15) As determined by MaHTAS, there was no evidence to suggest that the use of a disinfection box, chamber, tunnel, booth, partition, or gate could reduce COVID-19 infection, as the 20- and 30-second application process was insufficient for disinfection, and the chemical used in this process may be harmful if in contact with the eyes or mouth. (16) MaHTA’s evidence-based analysis of the technology addressed the misinformation and helped to create public awareness about the importance of evidence-based information.

MaHTAS emerged from the experience of COVID-19 as an agency able to achieve a new level of responsiveness. A transformation that otherwise might have taken a few years actually occurred in only a few months during the pandemic. MaHTAS worked within the limitations it had and re-asserted itself as an influential HTA agency that could derive new knowledge through collaboration and take on a central role in countering the COVID-19 infodemic.

## Story 3: How HTA helped Ontario families get answers: a genome-wide sequencing story

Unexplained developmental disabilities and multiple congenital anomalies are considered rare conditions. Although each rare condition affects only a small number of people, collectively they affect about 6–8 percent of the population. (17;18) Unexplained developmental disabilities and multiple congenital anomalies are difficult to diagnose, given their complex and overlapping symptoms, and about half of all congenital anomalies cannot be linked to a specific cause or diagnosis based on clinical presentation and environmental factors alone. (19) As a result, people with unexplained developmental disabilities or multiple congenital anomalies often spend many years seeking a diagnosis and undergo many diagnostic tests and procedures, commonly referred to as the “diagnostic odyssey.” (20) The lack of a diagnosis causes extreme stress for patients and families. (20).

For rare conditions with a suspected genetic cause, genome-wide sequencing (GWS) is the most comprehensive test for diagnosis and is used particularly when traditional genetic testing approaches (e.g., single-gene tests or targeted gene panels) have failed to identify or rule out a diagnosis. GWS examines the entire genetic makeup of a person in a single test and can be conducted as whole-exome or whole-genome sequencing. A genetic diagnosis can be key to understanding the cause and expected progression of a condition, avoiding unnecessary testing, and facilitating appropriate support systems for patients and families. (21).

In 2019, Ontario Health conducted an HTA (22) to inform a recommendation about publicly funding GWS in Ontario (Canada’s most populous province). (23) At the time, no Ontario laboratory was licensed to perform GWS as a clinical test for patient care, although some large academic centers were conducting GWS for research purposes. Access to GWS was limited, and the test was funded only through the Province’s Out of Country Prior Approval Program, which requires case-by-case approval. Eligible patients often waited six months or longer for testing to be completed, and little was known about the costs, quality, or outcomes of the testing. Additionally, guidelines on the use of GWS were unclear regarding the optimal timing to offer the test in the clinical care pathway.

To answer these questions, the HTA evaluated the clinical evidence of GWS on diagnostic yield and clinical utility through a systematic review and pooled-effect estimate calculations of the clinical literature and assessed the economic evidence on cost-effectiveness and potential budget impact. Importantly, the HTA also evaluated the experiences, preferences, and values of people with unexplained developmental disabilities or multiple congenital anomalies and those of family members. Patients and family members were interviewed to understand the lived experiences of patients’ conditions and care journeys. In addition, published quantitative and qualitative evidence on patient, family, and provider preferences and values was reviewed. An ethics analysis was also conducted to identify and reflect upon key ethical concerns related to GWS.

The HTA found that GWS has a higher diagnostic yield than standard genetic testing and, for some who receive a diagnosis, prompts changes to medications or treatments and facilitates specialist referrals. Direct patient engagement found that patients and families in Ontario want to receive a diagnosis through genetic testing and greatly value the support and information they receive through genetic counseling when considering GWS and learning of a diagnosis. The economic analysis showed that using whole-exome sequencing as a second-tier test (after the first-tier test, chromosomal microarray, fails to result in a diagnosis) would be the most efficient use of resources, being cost-saving and resulting in more diagnoses.

After reviewing the findings of the HTA, Ontario Health, based on guidance from the Ontario Health Technology Advisory Committee (24), recommended publicly funding whole-exome sequencing as a second-tier test.

The HTA report was posted online in 2020, and the HTA program routinely collects data to monitor its impact. As of September 2023, the HTA report had 1,183 page views and 373 downloads on the website and has been cited by other journal articles. The cost-effectiveness analysis was published in the journal *Genetics in Medicine*, which further disseminates HTA findings to the research and clinical communities.

The HTA and associated recommendations provided the foundation for an implementation project led by Genome-wide Sequencing Ontario (25) to offer GWS to Ontario patients suspected of having a genetic component to their otherwise unexplained condition(s). This project has helped Ontario establish a GWS program for the long term. As of 1 September 2023, 5,394 people in Ontario have received GWS (including 2,085 patients and 3,309 family members), 29 percent of families have received a clear or partial diagnosis of their child’s condition, the test has been repatriated (no longer to be sent to another country for administration) into local laboratories and, as a result, turnaround time has been reduced by half. For patients and families who received a diagnosis from GWS, this information has offered much needed support and the opportunity to connect with relevant worldwide social support groups, clinical specialists, and emerging treatments. Interestingly, the real-world diagnostic yield is very similar to the findings of the HTA, which estimated a yield of 34 percent for whole-exome sequencing based on the published clinical evidence.

Ontario Health’s HTA has led to the successful implementation of a new GWS program, which is now helping many Ontario families get answers and pursue appropriate care. HTA will continue to play an important role in the lifecycle of GWS, as this technology is quickly evolving and can be used to diagnose many other conditions.

## Story 4: Lifecycle health technology assessment and real-world evidence for high-cost medicine in Taiwan

This story describes the impact of Taiwan’s HTA on its reimbursement and health technology reassessment (HTR) policy for immune checkpoint inhibitors (ICIs), providing a perspective on improving the quality, consistency, and transparency of decision making.

ICIs are a major advancement in cancer treatment, but their cost-effectiveness remains uncertain, resulting in financial risk for the National Health Insurance Administration (NHIA). Taiwan started conducting HTA in 2007 to support the NHIA’s coverage decision making, focusing mainly on the introduction of new drugs into the NHI system. The Ministry of Health and Welfare (MOHW) authorized the Division of HTA in the Center of Drug Evaluation, Taiwan (CDE/HTA), to support the HTA program (26). Following the NHIA’s request, in 2017 the CDE/HTA reviewed the managed entry agreements (MEAs) programs of other countries implemented between 2015 and 2017, and it conducted clinical and economic assessments of four ICIs to facilitate policy making (27).

In 2017, through the INAHTA Listserv, HTA agencies that were members of INAHTA shared various experiences, noting that MEA programs were one of the most common approaches for dealing with high-cost treatments (27). By referencing other INAHTA members’ experiences and conducting a series of stakeholder meetings and communications in 2018, the NHIA adapted the MEA mechanisms to cover ICIs and proposed a set of general rules for reimbursement of high-cost drugs. As part of this scheme, the NHIA collects and assesses real-world evidence such as case registration data to adjust the benefit packages for each medication, to increase payment of benefits related to ICIs, and to present opportunities for improved NHI sustainability.

The adapted MEA scheme was a nationwide, multicenter, retrospective cohort study that assessed the real-world utilization, effectiveness, and safety of ICIs reimbursed by the NHI for treating multiple advanced cancers in Taiwan. Real-world data and real-world evidence were collected from the National Immune Checkpoint Inhibitor Registry Database developed by the NHIA. Real-world data and evidence from a certain period after NHI reimbursement will be collected and evaluated by the CDE/HTA.

Between 1 April 2019, and 31 March 2020, a total of 1,644 patients received at least one dose of ICIs. The overall response rate to ICIs was 29.1 percent of the total population. Patients with metastatic urothelial carcinoma who were ineligible for chemotherapy showed the highest response rates. The estimated median progression-free survival (PFS) was 2.8 months (95 percent CI, 2.7–3.0 months) in the total population (28). Based on the real-world evidence, the reimbursement policy for immuno-oncology drugs was updated in March 2020. Initially, three immuno-oncology drugs reimbursed by the NHIA—atezolizumab, pembrolizumab, and nivolumab—received extended coverage from one year to two years, excluding advanced or metastatic hepatocellular carcinoma (HCC) and metastasized gastric adenocarcinoma because of the relative lack of payment benefits in existing treatments. After failing to achieve risk-sharing agreements, the NHIA unprecedentedly suspended new applications in April (28).

In April 2021, the U.S. FDA Oncologic Drugs Advisory Committee (ODAC) opposed nivolumab for second-line advanced HCC and pembrolizumab for third-line indication in gastric/ gastroesophageal junction cancer (29). That decision is consistent with the results obtained by the CDE/HTA using real-world evidence one year later. Under the national registration tracking system, Taiwan’s high-cost drug policy has enabled access to new medicines and maximized patient benefits. To date, an evaluation of Taiwan’s reimbursement policy for such therapies indicates it is beneficial for patients, clinical personnel, manufacturers, and other stakeholders. In the coming years, close monitoring and evaluation will be required to analyze the effects of the current ICI treatment and the inevitable trade-offs between expenditures and improved patient access.

## Story 5: Enhancing uptake of a minimally invasive procedure for treating lower urinary tract symptoms of benign prostatic hyperplasia: the impact of a NICE recommendation and NHS funding schemes

Benign prostatic hyperplasia (BPH) is a noncancerous enlargement of the prostate that can cause lower urinary tract symptoms (LUTS). These symptoms include difficulty emptying the bladder, weak or intermittent urinary stream, and increased frequency of urination. The usual approach for managing LUTS associated with BPH is drug treatment followed by surgery if needed. BPH incidence increases with age, with an estimated increase from 50 percent of people with a prostate between the ages of 50 and 60 years, to 90 percent of those aged 80 years or over. (30).

NICE’s Medical Technologies Evaluation Programme (MTEP) identifies medical technologies that could offer a substantial benefit to patients or the health and social care system. After assessing the clinical and economic evidence, a decision is made on whether to recommend a technology for routine adoption in the NHS. This story describes the impact of MTEP’s evaluation of a prostatic urethral lift technology, a minimally invasive surgical option for reducing LUTS associated with BPH.

The technology’s journey through NICE began in 2014 with a recommendation from the Interventional Procedures program stating that the technology was safe and clinically effective (31). The technology was then evaluated by MTEP in 2015 to consider whether it offered value for money compared with current practice. The evaluation showed that the technology was clinically effective in relieving LUTS, while avoiding the risk to sexual function. Moreover, the procedure was cost-saving when compared to more invasive surgical procedures as it can be done as a day case rather than as an inpatient procedure, thereby reducing the costs associated with a hospital stay.

Despite MTEP’s positive recommendation in 2015, there was slow adoption of this technology in the NHS. This was in part because a positive recommendation from MTEP does not come with a legal obligation to fund the technology. Commissioners at individual hospital trusts make the decisions as to whether they use a NICE-recommended medical device. However, NHS funding and uptake schemes have been created to aid the increased adoption of selected innovative technologies where warranted. This prostatic urethral lift technology is an example of an MTEP recommendation supported by such schemes. It was added to the Innovation Technology Tariff in 2017 and selected as a Rapid Uptake Product by the NHS Accelerated Access Collaborative in 2018. Hospital episode statistics for prostatic urethral lift procedures in England between 2017 and 2020 showed an increase in uptake, with a total of eighty NHS trusts providing the procedure in 2020. (32).

During this time, a review of the 2015 guidance determined that an update to the evaluation was needed. The update, completed in 2021, utilized evidence from newly published randomized controlled trials and real-world evidence to demonstrate that the technology was still clinically effective and cost-saving in the recommended population, with longer-term studies showing that the technology could reduce LUTS for up to 5 years. (33;34) Six NICE shared-learning case studies also suggested that the technology was beneficial when used in the NHS, resulting in improved symptom and quality-of-life scores, reduced surgery times, and reduced hospital stays. (34) Additionally, the update expanded the recommended (or indicated) population to include people with BPH with an obstructive median lobe. The update also captured the growing trend of using this procedure in an outpatient setting, which would further reduce pressure on bed capacity. Following this update, the technology was added to the newly formed MedTech Funding Mandate, which aimed to accelerate equitable access to clinically effective, cost-saving medical technologies.

In addition to NHS funding support, the minimally invasive nature of the technology allowed for BPH procedures to be conducted in community hospitals during the COVID-19 pandemic. A case study from one NHS hospital trust demonstrated that this approach helped to reduce waiting lists and free theater and inpatient bed capacity in the main hospital for other procedures. (35) This effectively eliminated waiting lists for people eligible for the technology treatment who would have otherwise had to wait for a more invasive surgical procedure.

Overall, this case study demonstrates how continued data collection, including real-world data, can enable HTA bodies to broaden their recommendations. Moreover, it demonstrates how support from funding and uptake schemes can help drive routine adoption of procedures and devices that have otherwise had slow uptake following positive NICE medical technologies guidance.

## Story 6: Measuring the Impact of Health Technology Wales Guidance for autologous hematopoietic stem cell transplantation – one-year post-publication

Multiple sclerosis can be a highly disabling condition, having a significant impact on the quality of life for the person with the condition, their family, and carers. Symptoms are wide ranging, including visual and sensory disturbances, limb weakness, gait problems, and bladder and bowel symptoms. (36) Approximately 85 percent of people diagnosed with multiple sclerosis are diagnosed with relapsing remitting multiple sclerosis (RRMS), making it the most common type of multiple sclerosis. Over time, RRMS disability can get worse, and most RRMS cases develop into secondary progressive multiple sclerosis. Disease modifying therapies (DMTs) are used to treat RRMS, but for a small number of people DMTs stop being effective. Autologous hematopoietic stem cell transplantation (AHSCT) offers a potential alternative treatment option for people with RRMS where DMTs are no longer effective at controlling symptoms.

This story summarises the impact of Health Technology Wales (HTW) guidance on AHSCT for RRMS up to one year after publication, following an outcome evaluation process developed by the organization Matter of Focus. (37).

In 2019, the Welsh Health Specialised Services Committee (WHSSC) proposed AHSCT for RRMS as a topic for HTW appraisal. Following HTW’s rapid review process, the appraisal adapted and updated advice produced by the Scottish Health Technologies Group (SHTG). (38) HTW produced a *de novo* cost-utility analysis based on the key randomized controlled trial (39) comparing AHSCT with DMTs, which showed that AHSCT was dominant over DMTs. HTW subsequently recommended the routine adoption of AHSCT for people with RRMS, where symptoms have recurred despite previous treatment with DMTs. The HTW guidance and accompanying evidence appraisal report were published in July 2020. (40).

HTW engaged with stakeholders throughout the appraisal process and following the publication of the guidance. During the consultation period, the evidence appraisal report was shared with UK-based consultant neurologists, hematologists, lecturers, and professors. Other national HTA bodies, such as SHTG and the Irish Health Information and Quality Authority (HIQA) were also asked to review the report.

Following advice from the HTW patient and public involvement standing group, HTW sought engagement from two patient organizations as part of the appraisal process: MS Society Cymru and MS Trust. Both organizations provided independent patient submissions to reflect patient experiences and opinions. At the HTW Appraisal Panel meeting, a patient representative from MS Society Cymru gave a verbal account of their individual experience, including their experience of receiving AHSCT outside of the UK.

Following the publication of the guidance, positive feedback from both clinical and patient stakeholders was received. The guidance had been viewed online more than 480 times and was featured in multiple media articles. Patient groups welcomed the findings of the appraisal as an important step forward in recognizing the needs of people with RRMS, and the benefits of AHSCT.

At the time of sharing this story with INAHTA, HTW had undertaken a small pilot survey to measure the impact of its work. Those who responded said that the AHSCT appraisal and guidance had a major, positive impact on the wider health and social care context of Wales.

The Welsh Health Specialised Service Committee, which proposed the topic for HTW appraisal, reports that their prioritization panel had recommended AHSCT for RRMS as a high priority for funding in 2021 and that a WHSCC commissioning policy was in development.

This story demonstrates how using a structured evaluation process can help HTA organizations like HTW evaluate the impact of their work, and build a picture of the reach of HTA guidance and its influence through various measures.

## Discussion

The six HTA impact stories in this paper demonstrate many ways in which HTA can impact healthcare. The stories are as varied as the health system contexts they describe, with impacts observed on decision making, policy development, health services quality, and value for money.

The INESSS and MaHTAS stories tell of the sharp increase in demand for HTA from health system decision makers during the COVID-19 pandemic, which is an indication of the value of HTA as a trusted source of evidence and recommendations for health system decision making. HTA agencies faced demands for ultra-rapid HTA to support governments in making the well-founded decisions to manage the pandemic. The MaHTAS story describes their role in producing multiple, ultra-rapid assessments of the current state of evidence to inform government decisions about screening and treatment of COVID-19 as well as to repudiate misinformation that was circulating in the public domain about these technologies. The HTA work conducted during the COVID-19 pandemic was also an important catalyst for multistakeholder collaboration and alignment. The INESSS story tells of their review of Paxlovid^**MD**^ that aligned HTA, industry, and regulatory processes to provide rapid access for patients in need. These stories demonstrate that HTA production can accelerate beyond normal timelines to meet the urgent requirements of decision makers during the pandemic. Furthermore, the level of implementation response to decisions based on HTA evidence (and therefore HTA impact) was elevated, as most health systems provided free, immediate, universal access to technologies. In this case, the uptake and adherence to the implementation decision were supported by public mandates that are not typical outside of a public health emergency.

HTA is a policy tool to support health system decision making, and one indicator of HTA impact is the extent to which decision makers’ recommendations or directions align with HTA findings. The CDE story shows such an impact where changes to the policy were made in response to the HTA findings and results of a managed entry agreement. The Ontario Health story shows how the HTA report was used to support public funding for a genetic test that led to the establishment of a province-wide diagnostic testing program, thereby improving patient quality of life by shortening the diagnostic journey for many. The HTW account showed how that agency’s appraisal and guidance was based on a structured impact evaluation process and the major positive impact it has had in the wider health and social care context.

The stories also describe some of the challenges to achieving HTA impact. Jurisdictions that do not have a legal framework for HTA can experience slowed uptake and use of HTA findings as there is no supporting structure guiding or requiring the use of the HTA report. To help foster the use of HTA reports to improve health system quality and sustainability, some agencies provide support to those who are leading the implementation of the findings. The NICE and INESSS stories describe tools and techniques (e.g., prescription templates, continuing education, and ongoing data collection) utilized alongside the HTA to support the implementation and use of the findings in the health system. The provision of additional funding or in-kind support may be required to enable the health system decision makers seeking to use the HTA findings to implement the recommended changes and adjust accordingly to the use of any new care pathways or technologies. In addition, the HTW story shows how agencies can adapt and update advice from other trusted HTA bodies to rapidly and efficiently prepare reports to inform local decisions.

## Conclusion

HTA agencies evaluate the uptake, use, and effects of their HTA reports to understand what works well (or not so well) in achieving meaningful impact. The INAHTA impact story-sharing activity and the *David Hailey Impact Award* competition exemplify and advance the science and practice of HTA across diverse healthcare systems.

HTA agencies support governments and other decision makers in their use of evidence in decision making to improve health system quality and value for the populations they serve. INAHTA member agencies have shown their adaptability to the changing demands of decision makers through public health emergencies and other challenging circumstances, as well as everyday decisions about resource allocation. HTA agencies can provide timely, credible, transparent, evidence-based findings and recommendations in response to urgent and high-priority requests.

The importance of stakeholder involvement at key points in the HTA process was noted in most of the stories, with accounts of engagement with patients and clinical experts, as well as leveraged opportunities to align HTA processes with regulatory review. The insights derived from these stories expand and enrich the knowledge base for achieving and sustaining meaningful impacts of HTA.
